# Melodic Intonation Therapy for Post-stroke Non-fluent Aphasia: Systematic Review and Meta-Analysis

**DOI:** 10.3389/fneur.2021.700115

**Published:** 2021-08-04

**Authors:** Ana Haro-Martínez, Carmen M. Pérez-Araujo, Juan M. Sanchez-Caro, Blanca Fuentes, Exuperio Díez-Tejedor

**Affiliations:** ^1^Doctoral Programme, Department of Medicine, Universidad Autónoma de Madrid, Madrid, Spain; ^2^Brain Injury and Stroke Unit, Hospital Hestia Madrid, Madrid, Spain; ^3^Department of Medicine, Department of Neurology and Stroke Center, Hospital Universitario La Paz, Universidad Autónoma de Madrid, IdiPAZ Health Research Institute, Madrid, Spain

**Keywords:** post-stroke aphasia, speech and language therapy, melodic intonation therapy, meta-analysis, systematic review

## Abstract

**Introduction:** Melodic intonation therapy (MIT) is one of the most studied speech and language therapy (SLT) approaches for patients with non-fluent aphasia, although the methodological quality of the studies has been rated as low in previous reviews. The aim of this study is to update current evidence on the possible efficacy of MIT for the treatment of non-fluent post-stroke aphasia.

**Methods:** A systematic review and meta-analysis. We selected randomized clinical trials (RCT) that included adult patients over 18 years of age with non-fluent post-stroke aphasia, whose intervention was MIT vs. no therapy or other therapy. We excluded non-RCT studies, mixed populations including patients with aphasia of non-stroke etiology, studies with no availability of post-stroke aphasia-specific data, and incomplete studies. Three sections of communicative ability were analyzed as outcomes: functional communication, expressive language (naming and repetition), and comprehension.

**Results:** We identified a total of four eligible RCTs involving 94 patients. Despite the heterogeneity in the psychometric tests employed among the trials, a significant effect of MIT on functional communication (evaluated by the Communication Activity Log) was found (SMD 1.47; 95% CI 0.39–2.56). In addition, a positive effect of MIT on expressive language (repetition) was found (SMD 0.45; 95% CI 0.01–0.90). No significant effects on comprehension measurements were found, despite a lack of significant statistical heterogeneity.

**Conclusion:** This systematic review and meta-analysis shows a significant effect of MIT on improving functional communication and on repetition tasks. Future larger RCT specifically addressing those outcomes should provide the definite evidence on the efficacy of MIT on post-stroke aphasia recovery.

**Systematic Review Registration:**PROSPERO-URL https://www.crd.york.ac.uk/prospero/display_record.php?ID=CRD42020144604.

## Introduction

Aphasia is a disorder that is the result of an injury to the brain areas that control the production and understanding of language as well as its components (i.e., semantic knowledge, phonological, morphological, and syntactic). Aphasia is common after stroke, with an estimated frequency of 30 and 34% for acute and rehabilitation settings, respectively (1). Therefore, speech and language therapy (SLT) is currently seen as a key element in the rehabilitation of stroke as recommended by several scientific societies (2–4).

A Cochrane meta-analysis published in 2016 showed the effectiveness of SLT for post-stroke aphasia as compared to no therapy, in terms of better functional communication, reading, comprehension, writing, and expressive language (5). To date, there are several therapeutic approaches for patients with aphasia after stroke; among them, the most studied are constraint-induced aphasia therapy and melodic intonation therapy (MIT) (5). However, thus far, there has been insufficient evidence from comparative clinical trials to establish the benefit of one type of therapy over another (5). Therefore, the choice of one over other relies on the type and severity of aphasia and the experience and confidence of the therapist in each approach.

Melodic intonation therapy is a widely used therapy in clinical practice, and therefore, it is necessary to understand whether there is evidence of its efficacy. MIT has been proposed mainly for patients with significant defects in language production, poor verbal agility, poor repetition of sentences, exaggerated prosodic pattern of sentences, and relatively preserved auditory comprehension (i.e., mainly patients with non-fluent aphasia) (6–8). Patients with aphasia are trained to keep the rhythm of oral utterances that are initially sung by the therapist; the patient then tries to reproduce these statements while maintaining the prosodic pattern, intonation, and rhythm. As the therapy progresses, the therapist provides less support and the patient gradually suspends the rhythm and intonation until, finally, items are produced independently and with its usual prosody, being the final goal of MIT to restore propositional speech (7, 9). One of the advantages of MIT with respect to other SLTs is that it is a structured program that has been translated into several languages (10–14).

The mechanisms underlying the effects of MIT on aphasia recovery are not well-known, although it seems to stimulate brain plasticity by promoting the neuroplastic reorganization of language function, the activation of the mirror neuron systems, the utilization of shared features of music and language (such as pitch and rhythm) reflecting common or associated processing pathways, and improving the patient's motivation and mood (15). Indeed, some neuroimaging studies suggest effects of MIT on the stimulation of brain plasticity by activating language-capable regions of the right cerebral hemisphere and promoting left perilesional activation (9, 15–17).

A systematic review published 8 years ago reviewed the literature on the effect of musical elements in the treatment of patients with neurological language and speech disorders (18). The authors concluded that MIT was the most studied program in this field, although the methodological quality of the investigated studies was rated as “low” because they also included case studies and case series and the data were not meta-analyzed.

Our aim is to update current evidence on the possible efficacy of MIT for the treatment of non-fluent post-stroke aphasia in adult patients on functional communication, expressive language, and comprehension.

## Methods

### Search Strategy and Selection Criteria

This systematic review and meta-analysis is reported according to the Preferred Reporting Items for Systematic reviews and Meta-Analyses (PRISMA) recommendations for systematic reviews and the Cochrane guidelines for systematic reviews (19, 20). The study protocol has been registered in PROSPERO (ID CRD4202014460).

Our PICO (Population, Intervention, Comparator, Outcome) question to guide the systematic review was formulated as follows: in adult patients over 18 years of age with non-fluent aphasia due to ischemic stroke, does the MIT, as compared to no therapy or other therapy, improve functional communication, expressive language (naming and repetition), and comprehension?

The following databases were searched: Cochrane Central register of Controlled Trials (CENTRAL), PUBMED, EMBASE, Clinical trials gov. (http://clinicaltrials.gov/), and Clinical trials results (www.clinicaltrialresults.org). We also performed a manual search of reference lists in other prior systematic reviews on the same topic as well as in guidelines to identify further potentially eligible studies.

The terms used in combination for the search were: “aphasia,” “language disorder,” “stroke,” “post-stroke,” “speech or language therapy,” “melodic Intonation Therapy or MIT,” “randomized controlled trial,” “random allocation,” “controlled clinical trial,” “control group,” “double-blind,” “single-blind,” “cross-over studies,” “functional communication,” “communication evaluation,” “Boston Diagnostic Aphasia Examination,” "Western Aphasia Battery,” “Aachen Aphasia Test” “Communicative Abilities in Daily Living,” “Communicative Activity Log,” “Sabadel,” “Amsterdam Nijmegen Everyday Language Test.” The last literature search was performed on September 20, 2019 using neither language nor publication date restrictions.

We selected randomized clinical trials that included adult patients over 18 years of age with non-fluent aphasia due to ischemic stroke whose intervention was MIT vs. no therapy or other therapy. We excluded non-randomized clinical trials, those without a control group, inadequate randomization processes, mixed populations including patients with aphasia of non-stroke etiology, those with no availability of post-stroke aphasia-specific data, non-speech and language therapy studies, and incomplete studies.

### Data Collection and Analysis

The search results were merged with the reference management software (Mendeley Ltd). Duplicate records were deleted. Those separate reports from the same study were linked and evaluated as a single study. Studies analyzed in previously published systematic reviews were manually included when not obtained by the database search (5). Only published data were included in the review.

The evaluation of study eligibility was performed by two authors (AHM and CPA) with the supervision of the review coordinator who identified all potentially relevant articles. After examining the titles and abstracts, clearly irrelevant reports were discarded and the full text of potentially relevant reports was reviewed.

The following information was included in the data collection form: eligibility of the study and/or the reason for exclusion, study design, study duration, allocation and blinding process, possible sources of bias, total number of participants, study setting, diagnostic criteria, age, sex, relevant comorbidity, dates of the study, total number of intervention groups, specific interventions, outcome definitions, time-point of reported outcomes related to stroke onset, number of participants allocated to each study group, number of outcomes in each study group, missing data (lost to follow-up), summary data for each intervention group and outcome (2 × 2 table for dichotomous data; means and SD for continuous data).

For the analysis of the extracted data, we used the Review Manager 5 software (Version 5.3.5; Copenhagen: The Nordic Cochrane Centre, The Cochrane Collaboration, 2014).

### Quality Assessment and Bias Identification

The quality of the included studies and the risk of bias of each study were evaluated following Cochrane Collaboration recommendations available in the Cochrane handbook of systematic reviews of interventions: (20) sequence generation, allocation sequence concealment, blinding, incomplete outcome data, selective outcome reporting, and other potential sources of bias. Quality control and assessment of bias were performed independently by two authors. Disagreements were resolved by discussion until consensus was achieved.

### Interpreting Results and Drawing Conclusions

Under the coordination of the principal investigator, the entire team participated in this stage. Publication bias was assessed with the help of funnel plots. The results of the data analysis were imported into the GRADEpro Guideline Development Tool (McMaster University, 2015; developed by Evidence Prime, Inc.).

### Outcomes

Three areas of communicative ability were analyzed as outcomes: functional communication, expressive language (naming and repetition), and comprehension.

The main outcome was improvement in language skills or in functional communication constructs as measured by a formal evaluation with validated tools including the Boston Diagnostic Aphasia Examination (BDAE) (21), the Aachen Aphasia Test (AAT) (22), Sabadel (23), Amsterdam Nijmegen Everyday Language Test (ANELT) (24) and the Communicative Activity Log (CAL) (25). Naming, repetition, and comprehension were considered secondary outcomes and were measured by similar validated tools. In brief, the BDAE evaluates conversation and expository speech, auditory comprehension, oral expression, reading, and writing. The AAT evaluates six subtests including spontaneous language, repetition, auditory comprehension, and naming. The Sabadel mainly consists of a story retelling task that measures functional language. Finally, both the ANELT test and the CAL evaluate verbal communication in daily life.

### Statistical Analysis

Collected data for each outcome were mean and standard deviation after the treatment period as well as the number of participants in the experimental and control groups. Standardized mean difference (SMD) was the summary statistic chosen, given that it allowed for the comparison of various psychometric scales. Data were analyzed on a random-effects basis. Results were summarized as standardized mean differences (SMD) and 95% confidence intervals (CIs), and the results from tests evaluating the same outcome were pooled in forest plots for a more comprehensive analysis of the global effect across studies. Heterogeneity across the studies was evaluated considering clinical reasoning and statistical measurements such as the chi-squared and the *I*^2^ test. Sensitivity analyses were performed for each diagnostic tool to better identify possible sources of heterogeneity.

Finally, we used the GRADE approach to rate the quality of evidence, and we summarized the results in an evidence profile using the GRADE Pro tool.

## Results

### Database Search and Eligible Studies

**Figure 1:**
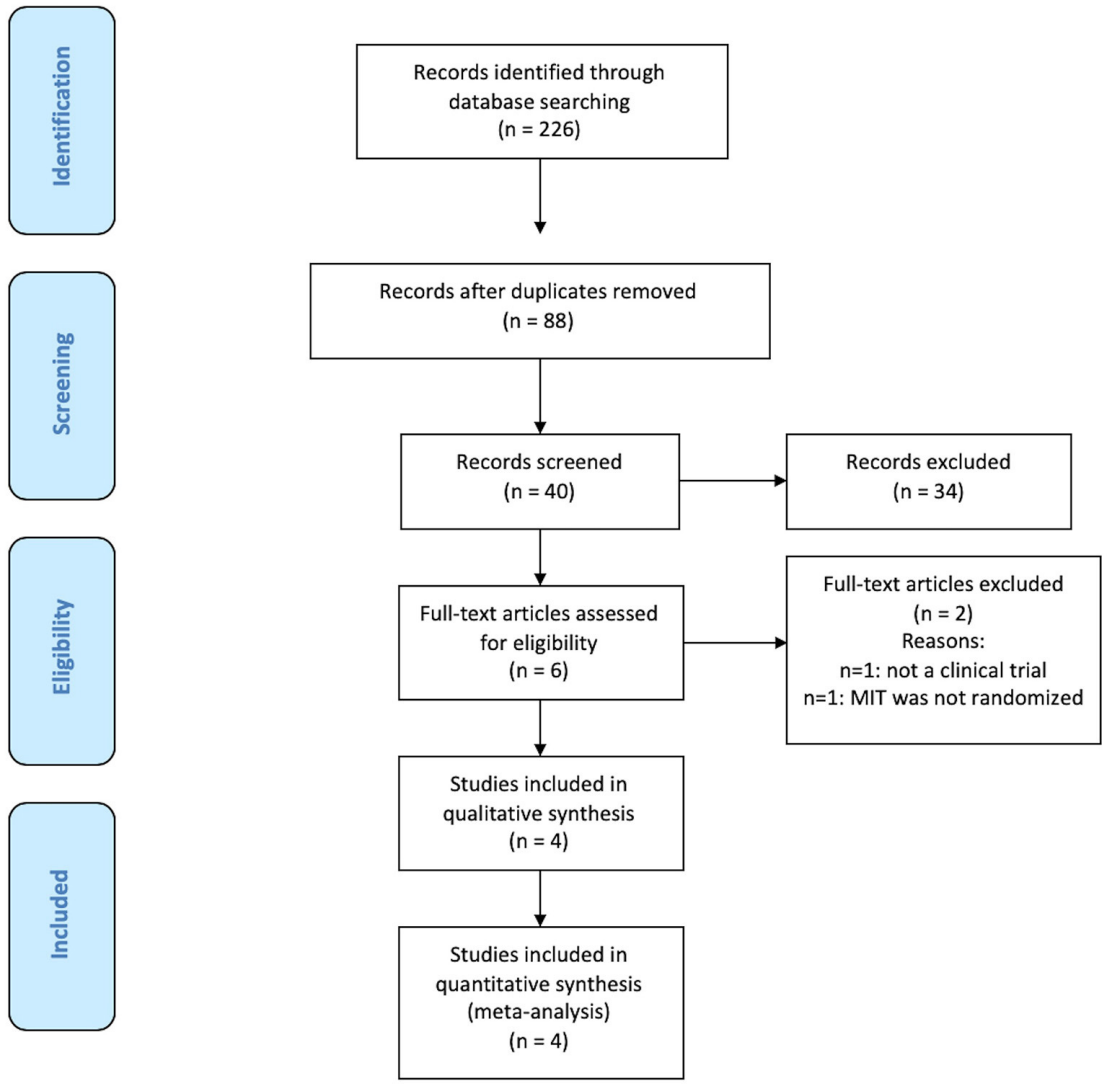
Flowchart of the selection of eligible studies.

Our search for articles in the databases produced a total of 226 results. After removing duplicates, 88 articles remained. The abstracts of these articles were analyzed, and 40 potentially eligible studies remained. Of those 40 studies, six studies were selected for full-text evaluation. Two were excluded: one for being a topic review (16) and the other because all the patients received MIT and they were randomized to transcranial direct current stimulation (tDCS) (26). Therefore, we included a total of 4 trials involving 94 patients in this systematic review (27–30) (Figure 1). Two studies were single, and two were multicenter and one of them used a modification of MIT (MMIT).

### Included Studies

Table 1 summarizes participant characteristics of the included studies. Conklyn et al. (27) was a pilot study with a randomized controlled design with blinded measurement of outcomes. A total of 30 acute stroke survivors with non-fluent aphasia were randomly assigned to receive MIT treatment or no treatment. They used a modification of MIT, the modified melodic intonation therapy (MMIT), in which the therapist has the freedom to modify the protocol by using novel melodic phrases that closely match the prosody of the spoken phrases in both pitch and rhythm, as well as full phrases during initial treatment. The aim of this approach is to develop individualized treatment plans and early stimulation of right brain language structures. Outcome evaluations were based on the differences between the pre- and post-test assessments of two tasks similar to the responsive and repetition subsections of the Western Aphasia Battery. These assessments were developed for the study, and were not, therefore, validated (27).

**Table 1:** Main characteristics of included studies.

	**Conklyn et al. (27)**	**Van der Meulen et al. (28)**	**Van der Meulen et al. (29)**	**Haro-Martínez et al. (30)**
Methods	Randomized, parallel group, single-blind, unicenter clinical trial	Randomized, waiting list, single-blind, multicenter clinical trial	Randomized, waiting list, single-blind, multicenter clinical trial	Randomized, crossover, single-blind, unicenter, pilot trial.
Main inclusion criteria	• 18 years of age or older • Mild to severe aphasia (score of 1 or 2 out of 3 on Item 9 on the NIHSS) • Damage to the left middle cerebral artery territory • No previous documented infarcts • Any dysarthria noted to be less than their aphasia • Ability to follow commands • Ability to sing at least 25% of the words of “Happy Birthday” • Demonstrated self-awareness of speech deficits	• Age 18–80 years • Aphasia after left hemisphere stroke • Time poststroke 2–3 months • Premorbid right-handed • Native language Dutch • MIT candidacy (non-fluent aphasia, articulation deficits, repetition severely affected, and moderate to good auditory language comprehension)	• Age 18–80 years • Right-handed before stroke • >1-year post stroke • MIT candidate • Native language Dutch • MIT candidacy: non-fluent aphasia after a unilateral left-hemisphere stroke, poor language repetition, poorly articulated speech, and moderate to good auditory language comprehension	• Age: no restrictions • Non-fluent aphasia due to unilateral stroke in the left hemisphere • > 6 months post-stroke • The patient had received a standard program of conventional speech therapy after stroke. • The patient had persistent non-fluent aphasia with the following characteristics: Severely restricted language, poor repetition, moderately preserved language comprehension
Intervention group	MMIT One to five sessions lasting 10–15 minutes	MITMinimum 3 h per week plus homework during 6 weeks	MIT Target: 5 h per week for 6 weeks (Minimum 3 h per week plus iPod-based homework)	MIT12 sessions over 6 weeks
Control group	No SLT	Waiting list (control intervention followed by delayed MIT)	Waiting list (control intervention followed by delayed MIT)	Waiting list (control intervention followed by delayed MIT)
Outcomes	• The responsive and repetition subsections of the Western Aphasia Battery developed for this study	• Sabadel • Amsterdam-Nijmegen Everyday Language Test • Aachen Aphasia Test (subtests repetition and naming) • MIT repetition task	• Sabadel story retell task • Amsterdam-Nijmegen Everyday Language Test • Aachen Aphasia Test (subtests naming, repetition and auditory comprehension) • MIT repetition task	• Communicative Activity Log • Boston Diagnostic Aphasia Examination
Participants	Total sample: 30 • MMIT group: 16 • Control group: 14	Total sample: 27 participants• MIT group: 16 • Control group: 11	Total sample: 17 • MIT group: 10 • Control group: *n* = 7	Total sample: 20 patients• MIT group: 10 patients • Control group: 10 patients
Main participant's baseline characteristics	Mean age: • MMIT group: 66.9 (SD 11.7) • Control group: 56.8 (SD 17.1) % Females: 46.6 Time poststroke, mean (SD): • MMIT group 32.2 (93.42) days • control group 28.4 (67.84) days,	Mean age:• MIT group: 53.1 (SD 12) • Control group: 52 (SD 6.6) % Females: 59.2 Time poststroke, mean (SD):• MIT group 9.3 (2.0) weeks • Control group 11.9 (5.9) weeks	Mean age: • MIT group: 58.1 (SD 15.2) • Control group: 63.6 (SD 12.7) % Females: 35.2 Time poststroke, mean (SD): • MIT group 33.1 (19.4) months • Control group 42.6 (23.7) months	Mean age:• MIT group: 66.9 (SD 14.7) • Control group: 61.1 (SD 14.1) % Females: 40Time from stroke onset, median (IQR):• MIT group: 21.8 (17.5) months • Control group: 27.7 (18) months
Comments	Missing data at main outcome visit: • MMIT group: 2 • Control group: 4	• Dropouts at main outcome visit: 3 • Loss to follow-up at main outcome visit: 2	No dropouts or loss to follow-up at main outcome visit	• Dropouts: 1 • Four patients allocated to control group crossed over to the MIT group, receiving the treatment first. • Loss to follow-up at main outcome visit: 1

*MIT, melodic intonation therapy; MMIT, modified melodic intonation therapy*.

Van der Meulen et al. (28) conducted a multicenter, waiting-list randomized controlled trial with a crossover design: patients were randomly allocated to the experimental group (MIT) or the control group (control intervention followed by delayed MIT) (28). A total of 27 participants were included: 16 in the experimental group and 11 in the control group. Outcome measures were the MIT repetition task, naming, repetition, and auditory comprehension subtests from the AAT; (22) the Amsterdam-Nijmegen Everyday Language Test; (24) and the Sabadel story retell task (23). The MIT repetition task comprised 11 trained and 11 untrained matched sentences.

Van der Meulen et al. (29) also used a multicenter waiting-list RCT design. Patients with chronic (>1 year) post-stroke aphasia were randomly allocated to the experimental group (6 weeks of MIT) or to the control group (6 weeks of no intervention followed by 6 weeks of MIT) (29). Assessments were performed at baseline (T1), after 6 weeks (T2), and 6 weeks later (T3). Efficacy was evaluated at T2 using univariable linear regression analyses. Outcome measures were chosen to examine several levels of therapy success: improvements in trained items, generalization to untrained items, and generalization to verbal communication. Of 17 included patients, 10 were allocated to the experimental group and 7 to the control group.

Haro et al. (30) was a randomized, crossover, interventional pilot trial. Participants were stroke survivors with post-stroke non-fluent aphasia. Patients randomized to group 1 received MIT first (12 sessions over 6 weeks) followed by no treatment; the patients in group 2 started active treatment between 3 and 6 months after their inclusion in the study, serving as waiting list controls for the first phase. Main measures were the CAL questionnaire and the Boston Diagnostic Aphasia Examination (BDAE), evaluated at baseline and at 6 and 12 weeks (21, 25). Twenty patients were included. Four of the patients allocated to group 2 crossed over to group 1, receiving the treatment first.

### Risk of Bias in Included Studies

The risk of bias assessments are summarized in Supplementary Figures 1A,B. The risk of selection bias was considered to be low; all articles used a computer-generated allocation sequence or a randomization table. Allocation was correct as well; two studies used consecutively numbered sealed opaque envelopes (28, 29) and in the other two studies, the patients were consecutively allocated as long as they were included in the trial (30) or allocation was performed by a nursing manager who had no prior knowledge of the order of participants (27). Performance (participant/personnel) bias was considered to be low in one study (27) and unclear in three studies (28–30). Detection bias was considered low in three studies (blinded measurement of outcomes) (27, 29, 30) and was unclear in one given that the authors acknowledged that blinding could not be maintained because the patients spontaneously informed the researcher about their therapy allocation (28). Attrition bias was considered to be low in three studies (intention to treat analysis) (28–30) and unclear in the fourth, given that some participants had incomplete or missing data and reasons for withdrawal were not reported (27). Finally, reporting bias was low in two studies and unclear in other two, given that not all of the prespecified outcomes were reported (27, 29).

**Figure 2:**
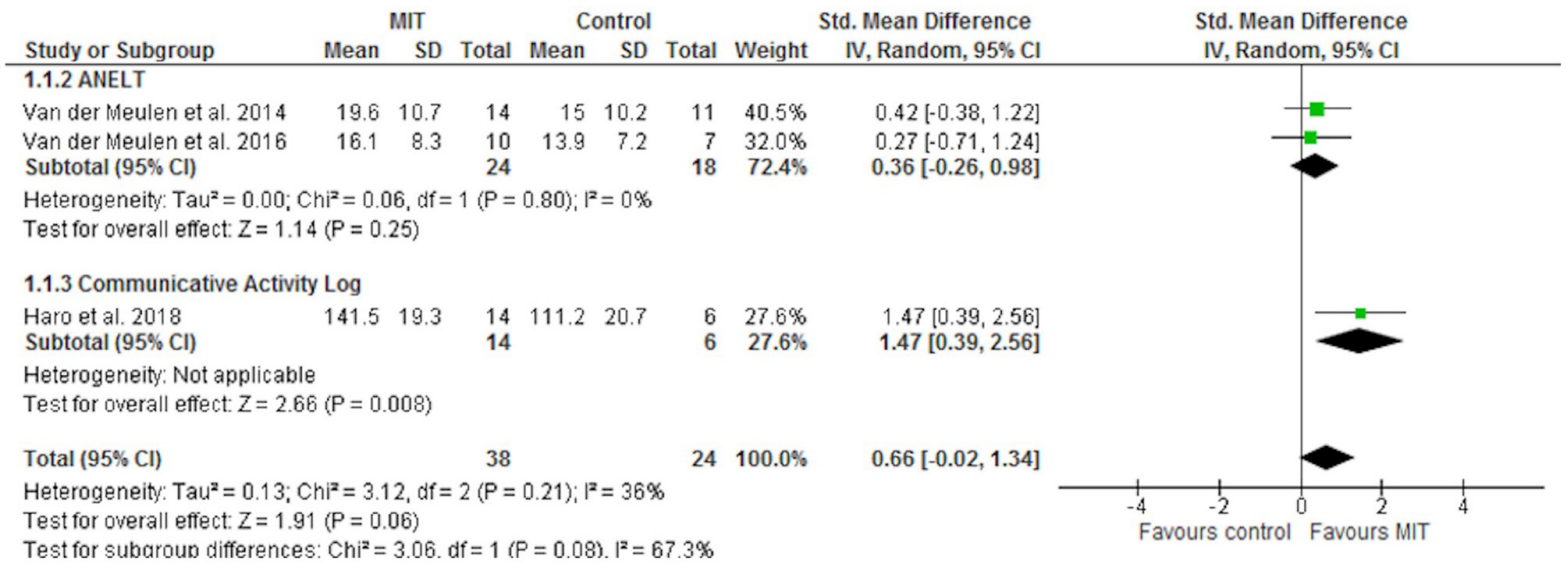
Forest plot of the effect of MIT on functional communication.

**Figure 3:**
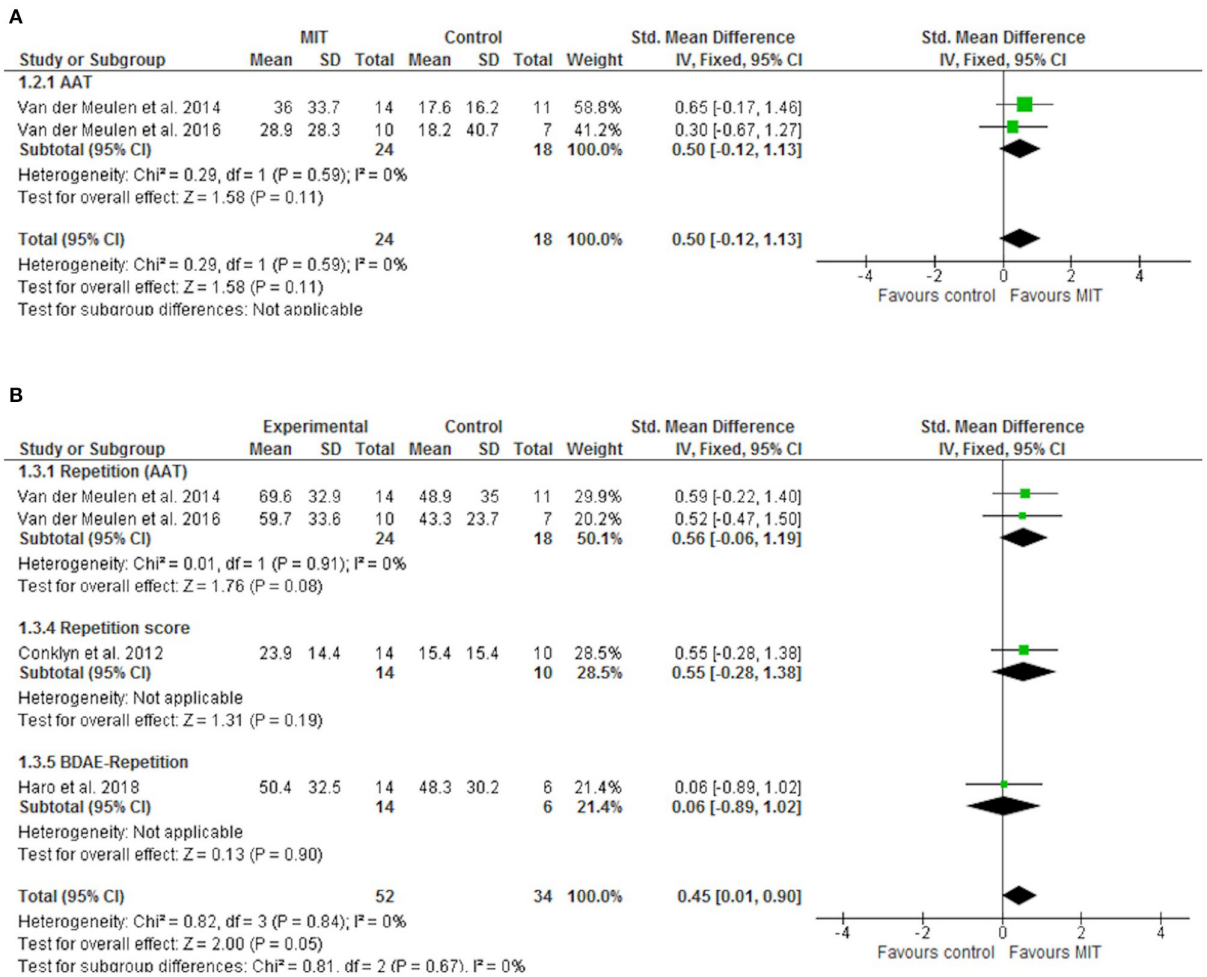
Forest plot of the effect of MIT on expressive language. **(A)** Naming; **(B)** Repetition.

**Figure 4:**
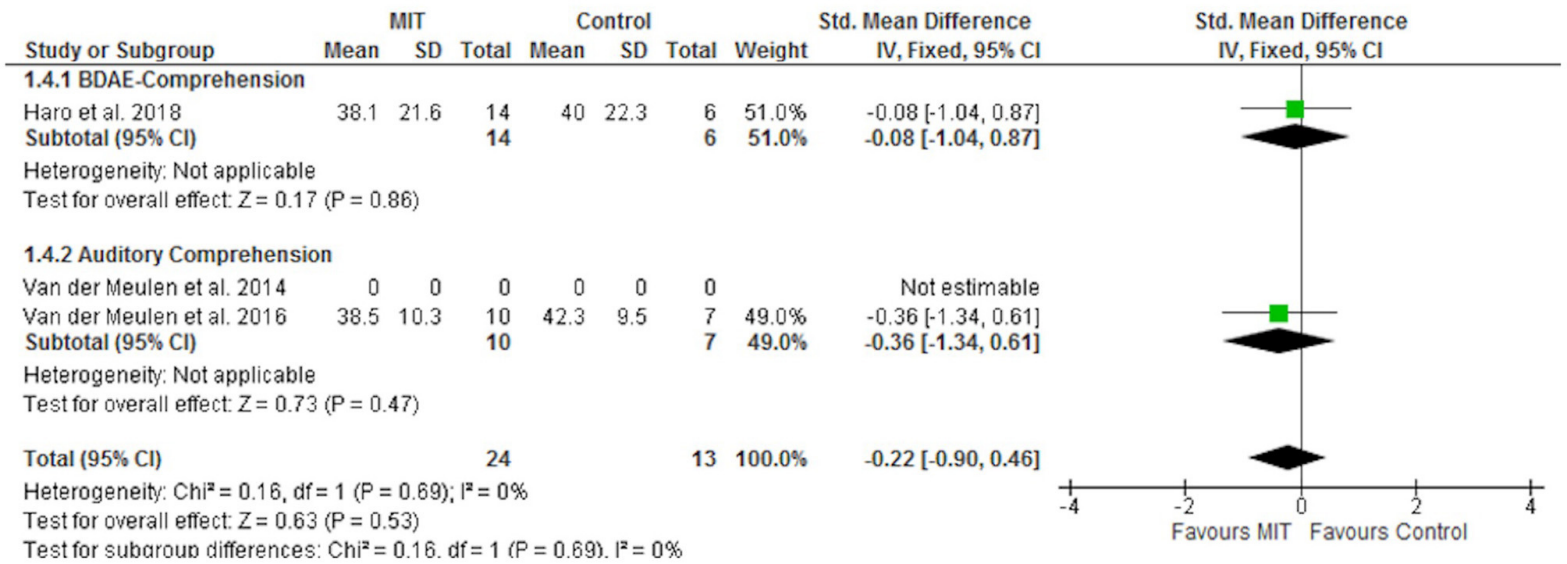
Forest plot of the effect of MIT on comprehension.

Figure 2 shows the effect of MIT on functional communication. Only the trial evaluating the CAL showed a significant effect of MIT on this outcome (SMD 1.47; 95% CI 0.39–2.56). Moderate heterogeneity was identified for this outcome (I2 36%). The main source of heterogeneity identified was the psychometric test chosen according to the sensitivity analysis performed (I2 67%). Besides, a positive effect of MIT on expressive language (repetition) was also found (SMD 0.45; 95% CI 0.01–0.90) (Figure 3). However, no significant effects on comprehension measurements were found (Figure 4). Neither of the secondary outcomes assessed showed significant statistical heterogeneity.

Supplementary Figures 2–4 show the funnel plots for each outcome evaluated in the meta-analysis. Supplementary Table 1 shows the summary of findings according to the GRADE criteria for evaluating the quality of evidence.

## Discussion

This systematic review and meta-analysis, compared to the 2016 Cochrane review which included only one RCT on MIT involving 27 patients, (5) provides information on 3 more published RCTs on MIT, involving 67 more patients (27, 29, 30). It shows a significant effect of MIT on improving functional communication (when evaluated by the Communicative Activity Log) and on repetition tasks. The global effect on functional communication shows the highest heterogeneity among the outcomes considered, which hinders statistical significance despite large effect sizes.

Research on post-stroke aphasia faces two main challenges that limit the internal validity of this study: (1) scarcity of published studies that meet the high standards of well-designed clinical trials, leading to a low number of included studies for the present meta-analysis; (2) heterogeneity in outcome measurements. In this meta-analysis, four randomized clinical trials were included and only two of them, conducted by the same research group, used the same endpoints. Due to the heterogeneity in the outcome measurements of the included studies, in those studies in which several outcomes were described, the most appropriate test for each of the outcomes considered in this review was chosen. Thus, for the functional communication outcome, the ANELT test was chosen over the Sabadel for the Van der Meulen et al. (28) and Van der Meulen et al. (29) studies, given that the ANELT showed less statistical dispersion and both are similarly validated tools. Concerning the repetition outcome, the AAT was chosen over the MIT-repetition trained items and MIT-Task untrained item tests, given that the latter are not well-validated tools (28, 29).

Functional communication represents the ability to successfully communicate in daily interactions, which should be the main goal for patients with post-stroke aphasia to ensure their social reintegration. However, less than half of clinical trials on SLT have focused on functional communication (5). In recent years, several initiatives have been developed to incorporate the perspectives of patients and their relatives into the definition of the most valuable outcome measurements for clinical trials, and research on aphasia has also followed this pathway (31, 32). Interestingly, both people with aphasia and their family members have rated improved communication as more desirable than other outcomes such as life participation or improved physical and emotional well-being (31). Other stakeholders such as clinicians and researchers also noted the relevance of communication as an activity/participation marker for evaluating patient recovery from aphasia (32). The Research Outcome Measurement in Aphasia (ROMA) consensus statement of 2018 recommended a set of outcome measurements for research in aphasia treatment (33). This was an important initiative and a first step toward a core outcome set for research in aphasia. However, the only instrument related to language was the Western Aphasia Battery Revised (WAB-R). The other recommended instruments belonged to the emotional well-being and quality of life domains, without consensus on a specific measure of communication (33). Among those communication tools evaluated by the ROMA panel were the CAL and the ANELT, which were used in the clinical trials included in this systematic review, and the MIT was associated with a significant improvement in CAL and a trend toward improvements in the ANELT. The CAL is a questionnaire targeting everyday language and communication activities, which is given to the patients themselves or to their relatives (25, 34). It has the advantage of evaluating patient use of verbal language in everyday life, providing information on both the amount and quality of communication in real-world settings (25). The ANELT evaluates the understandability of the message and intelligibility of the utterances in various scenarios during an interview with the patient, but not in real-life situations (24).

SLTs can be considered complex interventions per the definition of the Medical Research Council Framework for the Development and Evaluation of RCTs for Complex Interventions (35). Post-stroke aphasia can be heterogenous in its clinical presentation, with various impairments and grades of severity in individual patients. SLTs are also heterogenous in their approach and there are many outcome measurements for research on aphasia, therefore limiting the interpretation of results. In addition, some other factors that could impact the result of any SLT are individual patient factors (motivation, mental health status), family support (or lack thereof), and skill/experience of the clinicians. Systematic reviews of complex interventions can be problematic because the methodology of combining data from complex intervention studies is not yet fully developed (35). To reduce heterogeneity, we have conducted a systematic review focused on only one SLT, the MIT, and we included only randomized clinical trials. This approach has shown an improvement of the quality level of efficacy studies on MIT compared to the previous reviews (5, 9, 18).

The main limitations we faced were the small sample sizes in those trials as well as the heterogeneity in outcome measurements that prevented a pooled analysis. Nevertheless, we were able to show an effect of MIT on the CAL measurements and in the repetition tasks. Despite the randomized design of all the included trials and the lack of high-risk of bias, none of the clinical trials were sufficiently powered to demonstrate the efficacy of MIT. Therefore, the quality of evidence is moderate.

In conclusion, this systematic review provides updated evidence on the efficacy of MIT in improving functional communication and repetition in post-stroke non-fluent aphasia. Future larger RCT specifically addressing those outcomes should provide the definite evidence on the efficacy of MIT on post-stroke aphasia recovery.

## Data Availability Statement

The raw data supporting the conclusions of this article will be made available by the authors, without undue reservation.

## Author Contributions

AH-M and BF: conception of the work, acquisition, analysis and interpretation of data, draft of the manuscript, and approval of final version to be published. CP-A: acquisition, analysis and interpretation of data, draft of the manuscript, and approval of final version to be published. JS-C: analysis and interpretation of data, draft of the manuscript, and approval of final version to be published. ED-T: conception of the work, interpretation of data, and approval of final version to be published. All authors contributed to the article and approved the submitted version.

## Conflict of Interest

The authors declare that the research was conducted in the absence of any commercial or financial relationships that could be construed as a potential conflict of interest.

## Publisher's Note

All claims expressed in this article are solely those of the authors and do not necessarily represent those of their affiliated organizations, or those of the publisher, the editors and the reviewers. Any product that may be evaluated in this article, or claim that may be made by its manufacturer, is not guaranteed or endorsed by the publisher.
