# Supplementary material for: Melodic Intonation Therapy for Post-stroke Non-fluent Aphasia: Systematic Review and Meta-Analysis
Source: Front Neurol. 2021 Aug 4;12:700115. doi: 10.3389/fneur.2021.700115 (PMC8371046; doi:10.3389/fneur.2021.700115)
Supplement: Supplementary file 1 [file Data_Sheet_1.docx]

**SUPPLEMENTARY MATERIAL**

**Efficacy of melodic intonation therapy on non-fluent aphasia: A systematic review and meta-analysis**

**Supplemental Figure I:** Risk of bias summary

A) Authors' judgements of risk of bias items, presented as percentages across all included studies.


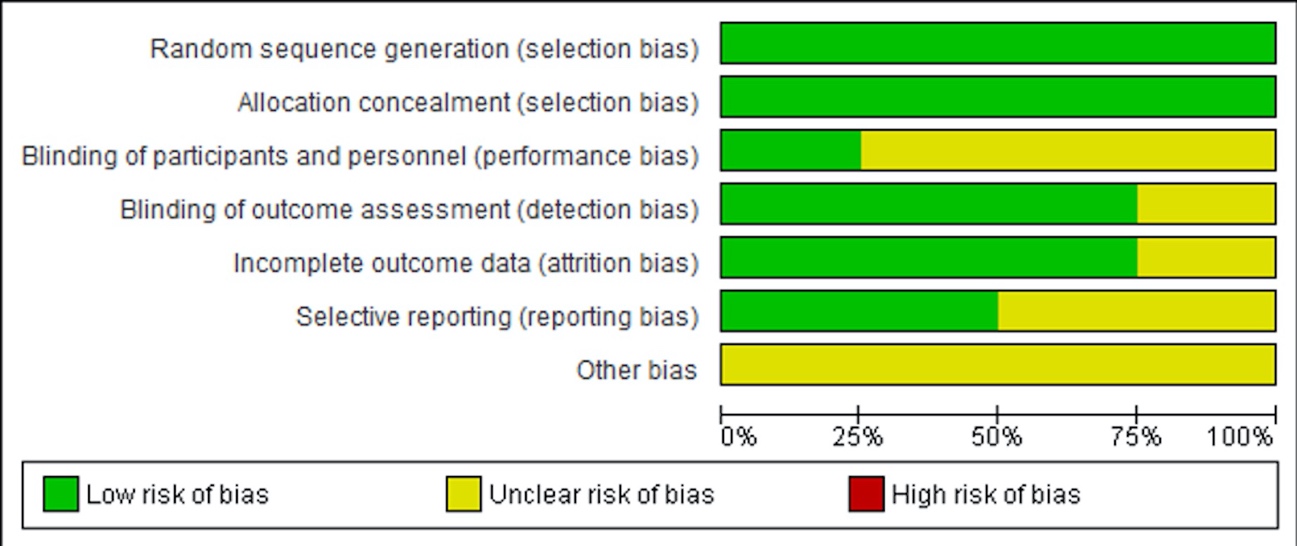


B) Authors' judgements of each risk of bias item for each included study.

**
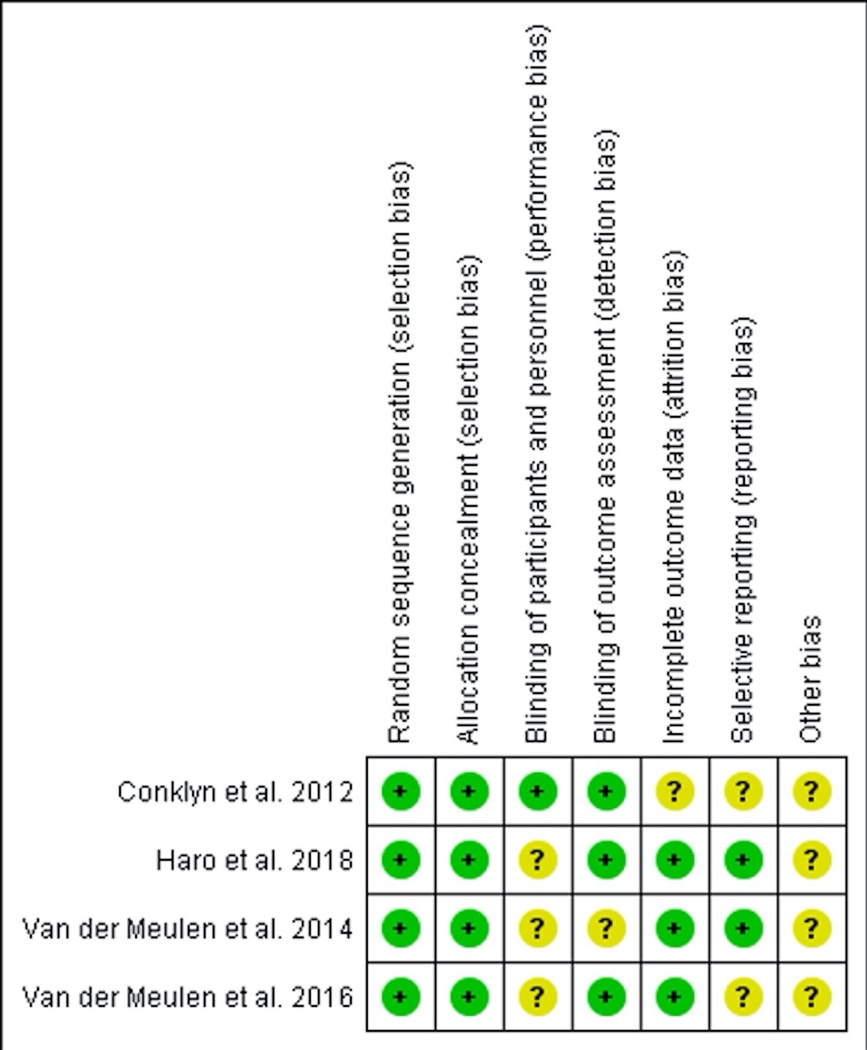
**

**Supplemental Figure II:** Funnel plots for functional communication.


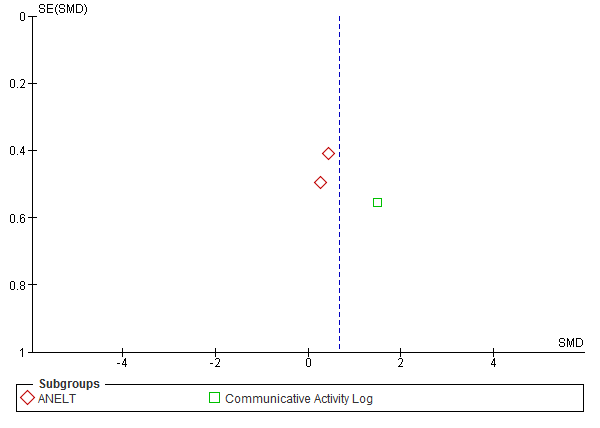


**Supplemental Figure III:** Funnel plots for expressive language.

1. Naming


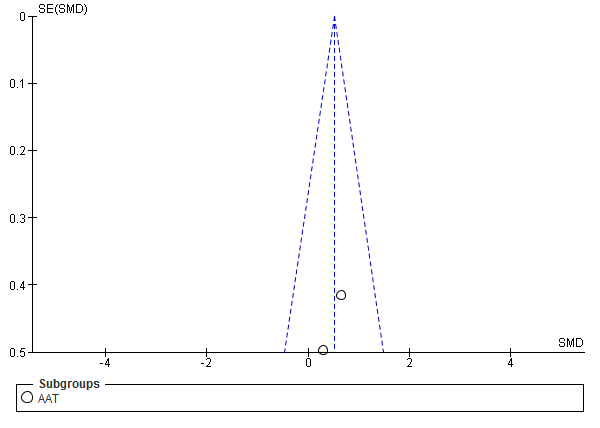


1. Repetition


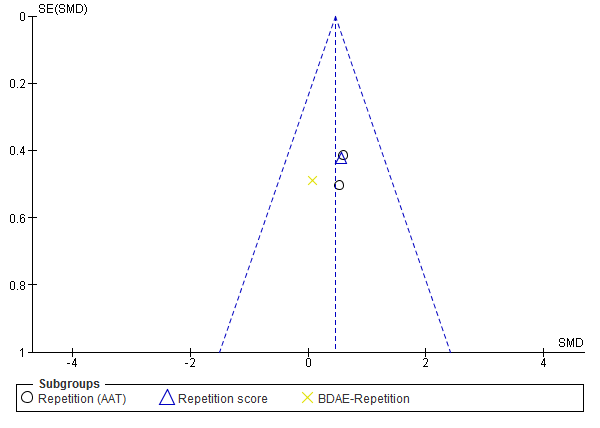


**Supplemental Figure IV:** Funnel plots for comprehension.


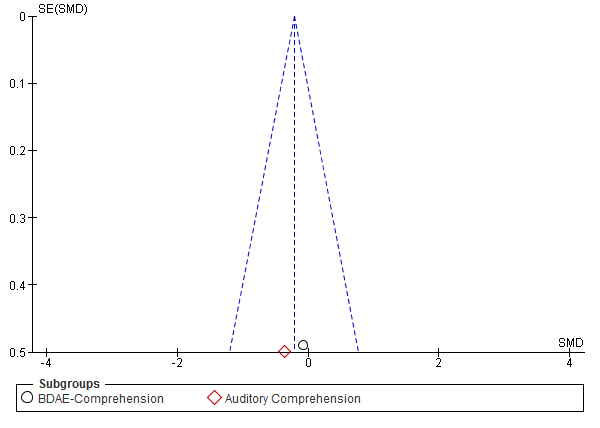


**Supplemental Table I:** Summary of findings.

| **Certainty assessment** | | | | | | | **№ of patients** | | **Effect** | | **Certainty** | **Importance** |
| --- | --- | --- | --- | --- | --- | --- | --- | --- | --- | --- | --- | --- |
| **№ of studies** | **Study design** | **Risk of bias** | **Inconsistency** | **Indirectness** | **Imprecision** | **Other considerations** | **MIT** | **no therapy or other therapy** | **Relative (95% CI)** | **Absolute (95% CI)** |  |  |
| **Functional communication** | | | | | | | | | | | | |
| 3 | randomized trials | not serious | serious ^a^ | not serious | serious ^b^ | none | 38 | 24 | - | SMD **0.66 SD higher** (0.02 higher to 1.34 higher) | ⨁⨁◯◯ LOW | CRITICAL |
| **Naming** | | | | | | | | | | | | |
| 2 | randomized trials | not serious | not serious | not serious | serious ^b^ | none | 24 | 18 | - | SMD **0.5 SD higher** (0.12 higher to 1.13 higher) | ⨁⨁⨁◯ MODERATE | CRITICAL |
| **Repetition** | | | | | | | | | | | | |
| 4 | randomized trials | not serious | not serious | not serious | serious ^b^ | none | 52 | 34 | - | SMD **0.45 SD higher** (0.01 higher to 0.9 higher) | ⨁⨁⨁◯ MODERATE | IMPORTANT |
| **Comprehension** | | | | | | | | | | | | |
| 3 | randomized trials | not serious | not serious | not serious | serious ^b^ | none | 24 | 13 | - | SMD **0.22 SD lower** (0.9 lower to 0.46 higher) | ⨁⨁⨁◯ MODERATE | IMPORTANT |

**CI:** Confidence interval; **SMD:** Standardized mean difference

#### Explanations

a. The Chi square test for heterogeneity shows a low P-value despite the low number of studies included. Furthermore, there is a highly likely subgroup effect contributing to heterogeneity. The I2 is moderate, also indicating overall heterogeneity.

b. CI intervals across studies show a fairly wide distribution crossing the clinical decision threshold due most likely to small sample sizes.
